# The Obstetric Catechism

**Published:** 1842-04-09

**Authors:** 


### BIBLIOGRAPHICAL NOTICES.

*The Obstetric Catechism.* By JOSEPH WARRINGTON, M.D. Philadelphia: 1842. pp. 340. 12mo.

The mode of instruction now adopted in many common and private schools and academies might be justly styled *the psittachian method;* a modern scientific school book, being too frequently a mere collection of heterogeneous questions, crudely and often incorrectly answered by the author in the succeeding paragraph. No exercise of intellect is required, either on the part of the preceptor or the pupil, to render one of these leather-cased budgets of fact and doctrine available; a good memory is amply sufficient. But, as to the real value of the knowledge derived from such sources, this may be admirably tested on almost any examination day by a very simple process. Take a station in front of one of the respectable files of intellectual puppets, fully trained by the careful teacher to astonish their parents and the assembly of strangers by the depth of their erudition, and while their examination is in progress, slyly translate a few of the stereotyped questions into equally lucid language, but with considerable variety of phrase. The experiment is somewhat cruel, or the anger and alarm of the learned instructor, and the utter consternation of the class of little automatic philosophers would be irresistibly ludicrous.

The general adoption of the system of *grinding*, as at present pursued in many medical schools, is exceedingly apt to produce similar results; especially when it is so ordered as to occupy the whole disposable time of the student in being drilled into the peculiar notions of a few professors, to the entire exclusion of the opinions of others;—an arrangement which necessarily deprives the pupil of the freedom of mind indispensable for the successful prosecution of scientific research, and often condemns him for life to judge all questions *in verba magistri.* The evils of this system are not confined to the student, but extend also to the professor, and react on the reputation of the school. The dignified occupant of a professorial chair, surrounded by those whose eyes and ears are thus closed against all knowledge from other sources, feels but a slender stimulus to exertion while secure in the assurance that at each succeeding annual ovation he will receive a salutation equally flattering with that reiterated by the thoroughly drilled Roman bird, "*Ave Cæsar, Imperator!*" But, fearing that we may be tempted into an aberration from the subject immediately before us, we will close this rather long introduction to a very short bibliographical notice with the simple remarks that, in the business of medical teaching, the near-sighted contemplation of immediate results so often disastrous to individuals, is equally dangerous to corporations; and that, in the management of great interests, it is often advisable to carry our calculations beyond the next election day. Were the merits of a professor or a school subjected to the judgment of a jury of graduates of ten years standing, the verdict would be found, in most cases, to differ very widely from that of the young class upon commencement day.

But the system of examination by stereotyped question and answer,—truly available only in the comparatively fixed sciences of anatomy and the materia medica proper, together, perhaps, with certain departments of chemistry—is so firmly established in the existing courses of medical instruction in most American schools, that it would be Quixottic to attempt its sudden overthrow. In the actual management of many of the subsidiary institutions, laudable efforts are made by the examiners so to regulate their labours as to exercise the reasoning faculties rather than the mere memory of the pupils, and also, to direct their attention to facts and opinions not exactly accordant with those of the collegiate teachers. And this is all that can be reasonably expected, unless the general features of medical instruction could be very essentially reformed.

The little volume before us is written on the purest plan of fixed and unalterable question and answer, but it has the advantage of being written by one unconnected with any collegiate school, and covers, so completely, the whole ground of the practice of obstetrics, that we may safely recommend it, not only to the pupils of the author, for whom it appears chiefly designed, but also to those young practitioners who desire a ready means of recalling the knowledge of facts connected with this important science which they may have previously acquired. Of its class, it is a well written book, and does much credit to the industry of its writer. If disposed to be hypercritical, some kindly fellow feeling for the egotistical would alone prevent us from smiling at the rather too fatherly mannerism of the preface, which, though not *very* censurable when assumed towards those addressed as "my own obstetric pupils," is hardly pertinent when extended to "students of medicine generally." A few pages are superadded at the conclusion of the work, in order to make known the arrangement of the author's obstetric study, the advantages of which are certainly worthy the attention of students desirous of knowing all the medical opportunities offered at the great medical school of Philadelphia. R. C.

